# Beaked whales demonstrate a marked acoustic response to the use of shipboard echosounders

**DOI:** 10.1098/rsos.170940

**Published:** 2017-12-13

**Authors:** Danielle Cholewiak, Annamaria I. DeAngelis, Debra Palka, Peter J. Corkeron, Sofie M. Van Parijs

**Affiliations:** Protected Species Branch, Northeast Fisheries Science Center, NOAA/NMFS, 166 Water Street, Woods Hole, MA 02543, USA

**Keywords:** echosounder, noise, beaked whales, detection rates

## Abstract

The use of commercial echosounders for scientific and industrial purposes is steadily increasing. In addition to traditional navigational and fisheries uses, commercial sonars are used extensively for oceanographic research, benthic habitat mapping, geophysical exploration, and ecosystem studies. Little is known about the effects of these acoustic sources on marine animals, though several studies have already demonstrated behavioural responses of cetaceans to shipboard echosounders. Some species of cetaceans are known to be particularly sensitive to acoustic disturbance, including beaked whales. In 2011 and 2013, we conducted cetacean assessment surveys in the western North Atlantic in which a suite of Simrad EK60 echosounders was used to characterize the distribution of prey along survey tracklines. Echosounders were alternated daily between active and passive mode, to determine whether their use affected visual and acoustic detection rates of beaked whales. A total of 256 groups of beaked whales were sighted, and 118 definitive acoustic detections were recorded. Regression analyses using generalized linear models (GLM) found that sea state and region were primary factors in determining visual sighting rates, while echosounder state was the primary driver for acoustic detections, with significantly fewer detections (only 3%) occurring when echosounders were active. These results indicate that beaked whales both detect and change their behaviour in response to commercial echosounders. The mechanism of this response is unknown, but could indicate interruption of foraging activity or vessel avoidance, with potential implications for management and mitigation of anthropogenic impacts.

## 1. Introduction

People have used sound to explore the marine environment for more than a century. Over the past few decades, our acoustic footprint in the oceans has dramatically increased, with noise in low frequencies now upwards of 20 dB higher than in the pre-industrial era [1]. Our growing contribution to noise in the ocean is increasingly putting humans into conflict with marine animals, many of whom rely on sound for basic life functions. Numerous studies have documented negative effects of acoustic disturbance, including physiological and behavioural changes, on a wide range of marine taxa. For example, vessel noise disrupts orientation and breeding behaviour in fishes [2,3], and it disturbs foraging and anti-predator behaviour and causes malformations during larval development in invertebrates [4,5].

Cetaceans are clearly sensitive to anthropogenic noise as well [6,7]. Behavioural responses vary from changing vocal behaviour to habitat avoidance (e.g. [8–10]), and physiological effects may include elevation in stress hormone levels [11]. Beaked whales appear to be particularly sensitive to anthropogenic sounds, with several well-known cases in which their responses to naval sonar have led to stranding and/or death [12,13]. Their sensitivity to other types of anthropogenic noise is not well understood, but they have been shown to change movement patterns and group behaviour in response to vessel noise at distances over 5 km [14]. Furthermore, the use of relatively low amplitude (135 dB re: 1 µPa SL) pingers operating at frequencies from 10 to 12 kHz on gillnets in fisheries in the Pacific Ocean eliminated bycatch of beaked whales [15], indicating that they both detected and avoided these acoustic signals.

Most of the aforementioned studies have focused either on the pervasive, low-frequency noise generated by vessel operation, or sound sources already known to be of concern, such as airguns or naval sonar. Little consideration has been given to one of the most ubiquitous sound sources in use today—commercial shipboard sonar systems (i.e. echosounders). Yet, behavioural responses by cetaceans to active commercial sonar systems, such as those used in fisheries and industrial activities, have been demonstrated in a few studies. Humpback whales (*Megaptera novaeangliae*) decreased their singing activity in response to a low-frequency fisheries sonar used during an experiment in the Gulf of Maine over 200 km away [16]. And off Madagascar, a mass stranding event of melon-headed whales (*Peponocephala electra*), in which an estimated 50 animals died, was probably triggered by the use of a 12 kHz multibeam sonar that was operated in association with seismic exploration [17]. Additionally, an experiment conducted with short-finned pilot whales (*Globicephala macrorhynchus*) demonstrated that they changed their movement behaviour when exposed to a commercial echosounder from a nearby vessel [18].

These observations are concerning, as commercial sonar systems are widely used by science and industry for fisheries and oceanographic research, benthic habitat mapping, and geophysical exploration (e.g. [19–22]), in addition to the traditional navigational and military uses. Countless ‘fish-finders’, lower-cost alternatives to scientific echosounders, are also in use on recreational vessels around the world. Furthermore, large-scale applications for active acoustics in ocean observation systems and for ecosystem-based management are being proposed, with conceptual frameworks covering numerous platforms (e.g. gliders, drifters, moorings, ships; [23,24]).

Many commercial sonar systems are categorized into one of three main classes: single-beam echosounders (SBES), side-scanning sonars (SSS) or multibeam sonars (MBES). Typical frequency ranges for these systems fall between 12 kHz and 400 kHz, with maximal source levels often ranging from 200 to 230 dB re: 1 µPa. While SBES are categorized by narrow apertures (typically 2–12°), with most energy concentrated directly below the ship, MBES may be configured with many beams spanning up to 150° or more. Additionally, omnidirectional sonars have become popular for long-range fish detection. Some models have a theoretical horizontal detection range up to 4500 m, transmitting in frequencies from 20 to 30 kHz with source levels over 215 dB [25]. Yet another system uses acoustic waveguide principles to preferentially propagate sound horizontally across the water column, which has been promoted for imaging fish schools over hundreds of kilometres [26].

Audiograms have been measured for at least 18 odontocete species, with frequency sensitivity ranging from a few kHz to over 130 kHz (for a review, see [27]). Empirical hearing threshold data are not available for mysticetes, but modelled data predict hearing sensitivities in the ranges from tens of Hz to approximately 20 kHz [28,29]. Many commercial sonars operate well within cetacean hearing ranges; even systems that are assumed to operate above the hearing ranges of cetaceans generate unintentional signals (i.e. side lobes) at frequencies that are audible to some species [30]. However, noise impacts are seldom considered in the operations of echosounders, despite their widespread use.

In an era of increasing human use of the oceans, it is imperative to better understand the potential impacts of commercial echosounders on acoustically sensitive species and how this influences their health, behaviour and survival. In this study, we conducted an experiment to determine whether beaked whales respond to a suite of Simrad EK60 echosounders, used for collecting standard scientific data. We treated as a single test group all beaked whale species which were potentially encountered during our study. These include Cuvier's (*Ziphius cavirostris*), Gervais' (*Mesoplodon europaeus*), True's (*M. mirus*), and Sowerby's (*M. bidens*) beaked whales. Echosounders were used during line-transect shipboard surveys to collect prey field data along survey tracklines. We evaluated whether visual and acoustic detection rates of beaked whales varied between days when shipboard echosounders were operated in active mode versus days in which they were operated in passive mode. We also tested whether the distribution of sightings varied between different echosounder states. Finally, we qualitatively evaluated the duration of acoustic events and the corresponding changes in bearing of acoustically tracked animals relative to the ship in the two different echosounder states.

## 2. Methods

### 2.1. Data collection

**Figure 1.**
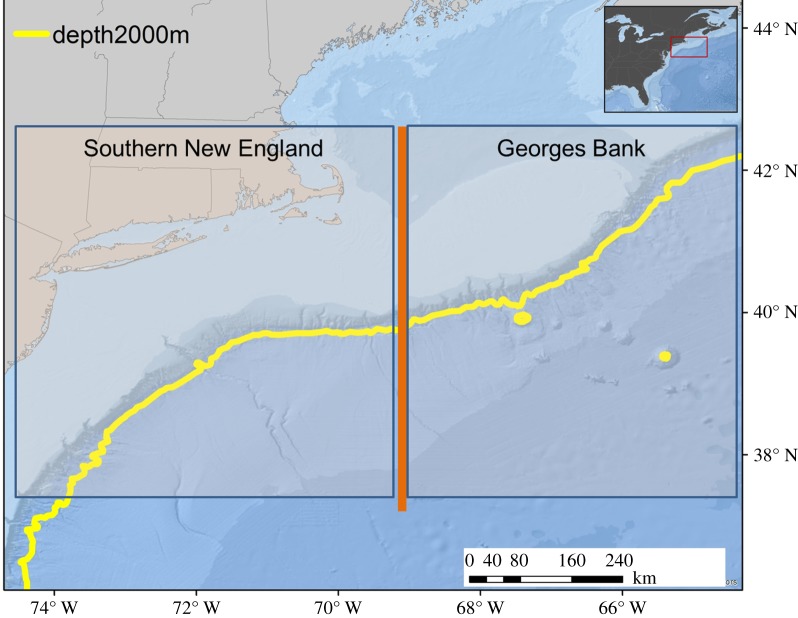
Study area, showing the habitat and region divisions used in the statistical analyses. The 2000 m bathymetric line (yellow) was used to divide the habitat between slope (less than 2000 m) and abyssal plain (greater than 2000 m). Region was broadly divided into southern New England and Georges Bank at a longitude of 69° W (orange line), which corresponds to the western edge of Georges Bank.

In 2011 and 2013, broad-scale cetacean assessment surveys were conducted as part of the Atlantic Marine Assessment Program for Protected Species (AMAPPS). The areas surveyed covered the waters east of North Carolina, US, to south of Nova Scotia, Canada (36°N to 42°N), and included approximately 5000 km of tracklines (figure 1). The surveys ran from 4 June to 1 August (2011) and 1 July to 19 August (2013), each divided into multiple legs. In both years, surveys were conducted from the NOAA research vessel *Henry Bigelow*, collecting data along the tracklines at speeds of 16–20 km h^−1^.

Visual data were collected by two teams of observers operating independently on separate decks of the vessel (15.1 m and 11.8 m above the sea surface). Each team consisted of four trained observers; two people used high-powered ‘big-eye’ binoculars (Fujinon, 25 × 150) to scan from the bow of the ship to 90° port or starboard, a centre person recorded data and scanned along the trackline using hand-held binoculars and the naked eye, with the fourth person being ‘off-effort’. Observers rotated between positions within their teams every 30 min. Visual data were collected during daylight hours from approximately 06.00 to 18.00 EDT when sea conditions were less than sea state Beaufort 6.

Data were collected on all marine mammals that were sighted. Sightings data included time, bearing and radial distance to sighting, species composition, estimate of group size, swimming direction, number of calves, and any additional observations. The survey was conducted primarily in ‘passing’ mode, though the ship did break track under certain circumstances to investigate groups of cetaceans when species identification was uncertain. Groups that were not identified to species were noted accordingly. The centre observer also recorded environmental conditions every 30 min, including visibility, glare, sea state, swell height and direction, and presence of rain, haze or fog.

Passive acoustic data were collected simultaneously with visual observations using a towed hydrophone array deployed 300 m behind the ship. The acoustic team consisted of three people who operated the system in 2 h shifts, collecting data during all hours when the visual team was on-effort, except along inshore tracklines where shallow bottom depths (50 m and less) prohibited safe deployment of the array, and a few periods when array maintenance was being conducted. The acoustic team also collected data on some occasions when weather conditions prevented the visual team from operating, as well as during several long transits between tracklines. Only acoustic data from the 2013 survey were analysed for this study, as the acoustic data from the 2011 survey suffered several compromises due to array failures.

The 2013 towed hydrophone array was comprised of two modular, oil-filled sections separated by 30 m of cable. The array included six APC International 42-1021 hydrophone elements (uncalibrated), two Reson TC 4013 elements (flat frequency response [±1.5 dB] from 1 to 180 kHz), custom-built pre-amplifiers (35–36 dB gain) and a depth sensor (Keller America, PA7FLE). Acoustic data were routed to a custom-built recording system that encompassed all signal conditioning, including analogue/digital conversion, filtering and gain. The recording system incorporated two National Instruments soundcards (NI USB-6356), one sampling the APC hydrophones at 192 kHz, the other sampling the Reson hydrophones at 500 kHz, both at a resolution of 16 bits. Data were high-pass filtered at 1000 Hz to remove flow noise, and an additional gain of 20–40 dB was added depending on the relative levels of signal and noise. Digitized acoustic data were recorded directly onto laptop and desktop computers using the software program Pamguard [31] (http://www.pamguard.org/home.shtml), which also recorded simultaneous GPS data and continuous hydrophone depth data. Array depth typically varied between 8 and 12 m. Sound speed data at the tow depth of the array were extracted from morning and midday CTD casts.

Active acoustic data were collected during the survey to characterize spatial distributions of zooplankton, fishes and other potential prey layers. Data were collected using Simrad EK60 single-beam scientific echosounders, operating simultaneously at the frequencies of 18, 38, 70, 120 and 200 kHz (table 1). Data were collected up to 3000 m bottom depth. The ping interval was set to 1 ping per second, but actual ping rate varied due to two-way travel time and signal processing requirements of the EK60. All EK60 transmitting frequencies were synchronized to reduce acoustic interference between instruments. In addition to the EK60s, an ES60 operating at 50 kHz was used by the ship's bridge for navigation.

**Table 1.** EK60 data acquisition parameters for the active acoustic data collected during the 2011 and 2013 cetacean abundance surveys on the R/V *Henry Bigelow*. ‘Absorption’ refers to the acoustic attenuation over distance. ‘Bandwidth’ is the receiver bandwidth setting. ‘Max. power’ is the maximum transmit power. ‘Beam width’ is the total angular width of the acoustic beam at the 3 dB (half-power) points. Table adapted from Jech [32].

	18 kHz	38 kHz	70 kHz	120 kHz	200 kHz
transducer type	ES18-11	ES38B	ES70-7C	ES120-7C	ES200-7C
absorption (dB km^−1^)	2.1	8.1	21.5	40.5	60.7
pulse duration (ms)	1.024	1.024	1.024	1.024	1.024
bandwidth (kHz)	1.57	2.43	2.86	3.03	3.09
max. power (W)	1000	1000	1000	500	300
beam width along/athwartship	10.9	7.0	6.9	6.5	6.26

For the purposes of conducting the current study, the EK60s were alternated between ‘active’ and ‘passive’ mode every other day during the surveys. The bridge fathometer was also alternated between modes whenever the ship's captain felt it was navigationally safe to do so. While in active mode, the echosounders transmitted acoustic signals; while in passive mode, the transducers received sound data but did not actively transmit signals. Visual observers were blind to EK60 state.

### 2.2. Data analyses

Visual data were post-processed using custom-built software to evaluate all sightings. Visual sightings of all beaked whales were extracted for analysis. Duplicate sightings between the two visual teams were removed, such that only one sighting per cetacean group was used in the analyses. If both teams sighted the same group, the sighting from the upper team was used. In some cases, groups were tracked for several minutes before the group dove; in these cases, data from the final surfacing were used.

Acoustic data were post-processed using Pamguard version 1.12.05 in a two-stage procedure. First, the Pamguard click detector was run over all sound files (software settings: pre-filter: 16–90 kHz; trigger filter: 20–90 kHz; threshold 13 dB). This produced a dataset with detections of click-like signals from multiple sources (cetacean echolocation, echosounder, and noise). Click detection data were then analysed by a trained acoustic analyst (AID) to identify putative beaked whale events. Data were browsed with a 5 min page window for days when echosounders were in passive mode. During the days when echosounders were active, data were browsed with a 2 min page window to ensure that events were not missed due to any potential interference from detections of the echosounder.

**Figure 2.**
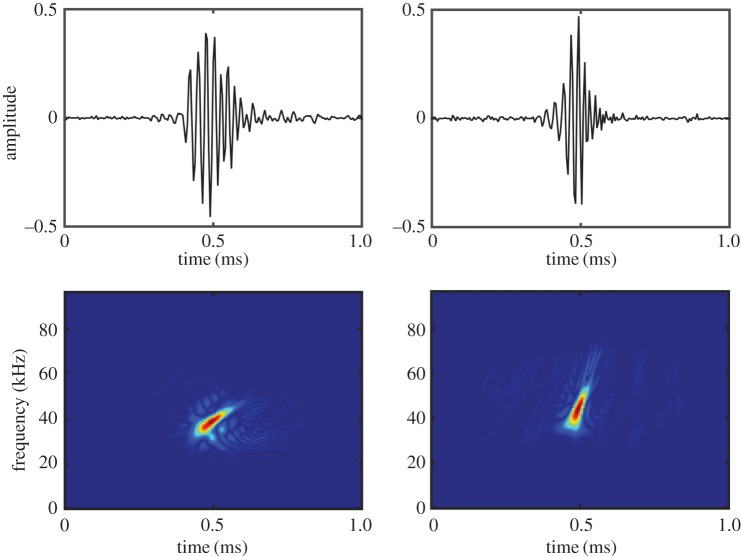
Example beaked whale clicks extracted from the 2013 towed hydrophone array data, showing the waveform (top) and Wigner–Ville spectrogram (bottom). The click on the left is from a Cuvier's beaked whale (*Z. cavirostris*), the click on the right is from either a Gervais' or True's beaked whale (*M. europaeus* or *M. mirus*).

The waveform, power spectral density (PSD), Wigner–Ville plot and concatenated spectrogram windows were used to assess if clicks had beaked whale characteristics. Clicks were considered putative ‘beaked whale’ if there was a bell-shaped envelope in the waveform, the majority of the energy between 30 and 70 kHz in the PSD, and an upsweep present in the Wigner–Ville plot (figure 2). Clicks were clustered into click trains if the inter-click interval (ICI) was between 0.2 and 0.6 s, as described for several species of beaked whales [33–35], and if clicks were in relatively the same bearing. Click train segments were grouped as an ‘event’ if they followed the same bearing line, or gradually changed in bearing over time. The concatenated spectrogram was reviewed to confirm that the distribution of energy of all clicks in events corresponded to published frequency bands of known beaked whale species[34,36,37].

Events were assigned to one of three categories, which were conservative by definition so as to minimize the chance of misassignment: (i) definite beaked whale (BEAK), in which an event had 10+ clicks, consistent ICI, and at least 5 clicks with upsweeps, (ii) probable beaked whale (PRBK), in which an event had more than 5 clicks, consistent or inconsistent ICI, and at least 3 clicks with upsweeps, or (iii) possible beaked whale (POBK), where an event had 1–5 clicks with a consistent ICI, with all clicks having upsweeps.

Determining the group size of acoustic events is difficult due to the nature of towed hydrophone array data and the directionality of beaked whale echolocation clicks. Due to the left–right ambiguity inherent in linear array data when the ship is transiting in a straight line, it is not possible to determine which side of the ship corresponds to an acoustic detection. Additionally, when multiple animals dive together, differences in bearing between animals become minimal at large ranges from the ship, and the directionality of beaked whale clicks makes it difficult to consistently track individuals. Therefore, while beaked whale detections were tracked at the individual level to the extent possible, some events may also represent multiple whales.

### 2.3. Statistical analyses

Only days in which shipboard tracklines covered shelf break and abyssal waters, both of which comprise potential beaked whale habitat, were included the analyses. Days in which the survey effort was focused in shelf waters less than 100 m deep were excluded.

Acoustic and visual events were grouped by day into three separate datasets: (i) visual sightings of groups from 2011 & 2013 combined, (ii) definite acoustic beaked whale events (BEAK) from 2013, (iii) all potential acoustic beaked whale events (BEAK, PRBK, POBK) from 2013. In each of the three datasets, only events that had a start time occurring during daylight hours (06.00–19.00 EDT) were used, to provide the best comparison between acoustic and visual data. For the purposes of this study, beaked whale events were not differentiated by species.

To test whether the number of detected beaked whale groups differed between days when the EK60 echosounders were operating in active mode, versus days in which they were operating in passive mode, a regression analysis using generalized linear models (GLM) was conducted using the statistical software R [38]. We included the following covariates: echosounder state (active versus passive), daily median sea state (low: 0–2; high: 3+), habitat type (slope or abyssal), and region (southern New England or Georges Bank). The daily median sea state was calculated based on the Beaufort sea state recorded by the visual team every 30 min when they were on effort. Since the visual team did not collect data in sea states of 6 or higher, a default value of 6 was used for those periods where only acoustic data were collected. Sea state was then divided into ‘low’ and ‘high’ categories, as beaked whale encounter rates are known to decrease drastically with sea state [39], and there were not enough data to support discrete categories. Habitat type was considered ‘slope’ if the majority of the ship's daily tracklines occurred on the shelf break between 100 and 2000 m, and ‘abyssal’ if the majority of the ship's daily tracklines occurred away from the shelf break in waters 2000 m or deeper. The survey area was also divided into two broad regions at a longitude of 69° W, which delineates the western edge of Georges Bank in the Great South Channel. To the east of this line, the Georges Bank region is defined by a large, submarine bank that forms a boundary with the Gulf of Maine, creating an area of frontal zones and high productivity. The southern New England region (SNE) was defined as the area west of 69° W. Daily trackline distance was used in the GLM to account for differences in daily effort. On days in which acoustic and visual teams were simultaneously on effort, the trackline distance was the same across all three datasets. On days in which the acoustic and visual effort differed, trackline distance was summed separately for each dataset.

A Poisson GLM was first applied to each of the visual and acoustic datasets, and overdispersion was assessed by applying a quasi-Poisson GLM. The data showed evidence of overdispersion in both visual and acoustic datasets, thus negative binomial GLMs were used in lieu of Poisson GLMs. Negative binomial GLMs using a log link function were run using the MASS package in R [38,40]. The link function is implemented in generalized linear models to relate the value of the response to the linear predictor of the explanatory variables; the log link function is appropriate for count data that follow a negative binomial distribution. Models were assessed with backwards stepwise selection, using Akaike's information criterion (AIC) to identify the best explanatory models [41]. The model with the lowest AIC value was considered the best approximating model [41]. Models were also assessed with the single-term deletion method using Chi-squared goodness-of-fit tests to select the model of best fit as a means of cross-validation. Model assumptions were checked to ensure that the relationship between the response and explanatory variables was linear on the function's link scale, observations were independent from one another, and there were no influential observations on the fit of the model. Variance inflation factors (VIF) were used to assess collinearity between variables. Finally, the phia package in R [42] was used to estimate adjusted mean values of sightings or acoustic events for interactions between variables in the most parsimonious model. Finally, to test whether the distribution of sightings differed between days with echosounders in active versus passive mode, radial distances were compared using a Kolgomorov--Smirnov test, which measures the maximum vertical deviation (*D*_mvd_) between the two distributions.

Definitive acoustic beaked whale events (BEAK) were qualitatively evaluated to determine whether there was a difference in event duration and bearing distribution when echosounders were in active versus passive modes. Quantitative tests were not used, as the sample size when echosounders were active was not large enough to allow for a robust statistical test.

## 3. Results

### 3.1. Survey effort

Across both years, 63 days of survey effort were included in the analyses of visual sightings. In 2011, the visual team surveyed 5047 km while on effort, of which 4119 km across 33 days were included in the analyses. In 2013, the visual team surveyed 5021 km, and a total of 3935 km across 30 days were included in the current analyses. Average trackline distance surveyed per day was approximately 128 km. Effort was similar between habitats and regions (table 2).

**Table 2.** Visual and passive acoustic trackline effort included in analyses. Slope habitat comprised tracklines that transited the shelf break, from approximately 100 to 2000 m depth. Abyssal habitat included tracklines beyond the shelf break, in water depths of greater than 2000 m. Shallow water (less than 100 m) tracklines were not included in analyses. Region was divided into southern New England (SNE, west of 69°W) and Georges Bank (east of 69°W). All trackline effort is reported in kilometres surveyed. Note that acoustic data were collected in 2013 at times when the visual team was not operating, leading to slightly higher survey effort.

	effort summary		km/habitat		km/region		km/echosounder state	
	days	total km	slope	abyssal	SNE	Georges	EK60 off	EK60 on
2011 visual	33	4119	2132	1987	2335	1784	2145	1974
2013 visual	30	3935	2158	1776	2124	1811	1916	2019
2013 acoustic	33	4968	2704	2264	2554	2414	2443	2524

During the 2013 survey, acoustic monitoring was conducted on 33 days, for a total of 271.88 h of daytime recording covering 4968 km of survey tracklines. The acoustic team collected data in some conditions that were prohibitive for the visual team (e.g. fog, heavy sea state), leading to greater total trackline coverage (table 2).

Daily median sea state in which visual surveys were conducted ranged from a low of Beaufort 1 (3 days) to a high of Beaufort 5 (3 days). The majority of the survey effort in both years had median daily sea states of 3 or higher (46/63 days; table 3). In 2013, limited acoustic survey effort was also conducted in Beaufort 6 conditions (table 4).

**Table 3.** Visual effort and sightings of beaked whales for the 2011 and 2013 surveys combined, compiled by sea state. Approximately 67% of tracklines were surveyed in ‘high’ sea states (3+).

	*EK60 passive*				*EK60 active*			
Beaufort category	# survey days	km surveyed	# groups sighted	groups/km	# survey days	km surveyed	# groups sighted	groups/km
1	1	165	47	0.3	2	348	21	0.06
2	5	857	60	0.1	9	1291	47	0.04
3	12	1623	31	0.02	10	1292	12	0.01
4	11	1132	16	0.01	10	1045	19	0.02
5	2	283	2	0.01	1	17	1	0.06
*sum*	*31*	*4060*	*156*		*32*	*3994*	*100*

**Table 4.** Acoustic effort and detections of beaked whales for 2013 survey, compiled by sea state and echosounder use. # BEAK indicates the number of definitive beaked whale detections.

	*EK60 passive*				*EK60 active*			
Beaufort category	# survey days	km surveyed	# BEAK	detections/ km	# survey days	km surveyed	# BEAK	detections/ km
1	1	170	6	0.04	1	204	0	0
2	1	201	15	0.07	6	1023	2	<0.01
3	4	559	9	0.02	4	467	1	<0.01
4	6	980	66	0.07	4	630	1	<0.01
5	2	328	16	0.05	1	86	0	0
6	2	205	2	0.01	1	114	0	0
*sum*	*16*	*2443*	*114*		*17*	*2524*	*4*	

Echosounder use was alternated nearly equally between active and passive states. In 2011, the EK60 was operated in active mode on 16 days and in passive mode on 17 days. In 2013, it was operated in active mode on 17 days (16 included in visual analyses) and in passive mode on 16 days (14 included in visual analyses). Trackline effort was also similar between echosounder states (table 2).

### 3.2. Visual sightings data

**Figure 3.**
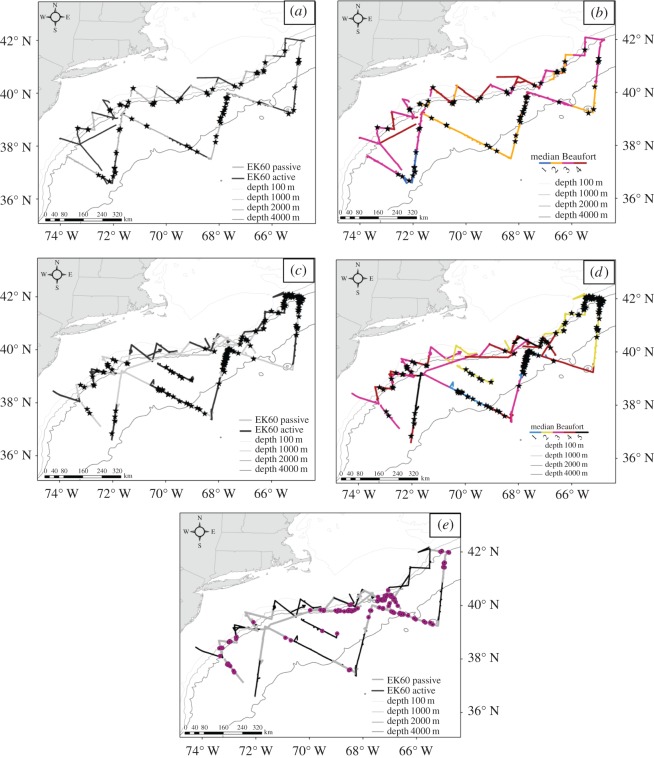
Map of tracklines surveyed in 2011 (*a*,*b*) and 2013 (*c*,*d*,*e*), by EK60 status and Beaufort sea state. Visual sightings of beaked whales are represented by black stars (*a*--*d*); acoustic BEAK detections are represented by purple circles (*e*). Note that the statistical analyses combined sea states into low (Beaufort 1–2) and high (Beaufort 3+).

A total of 256 groups of beaked whales were sighted between the two surveys, with an estimated total of 638 individuals (figure 3). Overall, 156 groups were sighted when echosounders were in passive mode, and 100 were sighted when echosounders were active.

**Figure 4.**
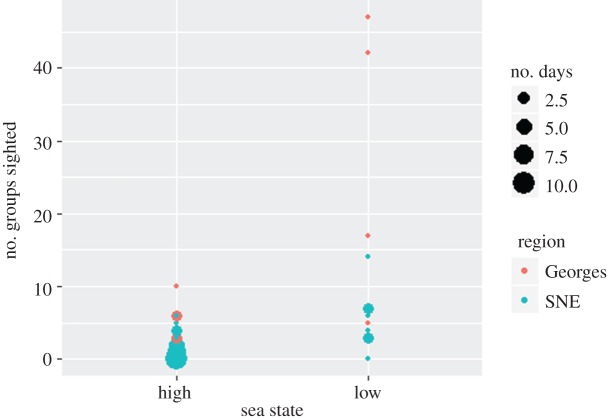
Number of beaked whale groups sighted per day, by sea state and coloured by region. Pink dots represent sightings in the Georges Bank region; blue dots represent sightings in the southern New England (SNE) region.

In 2011, 81 groups were sighted across 23 survey days, with similar proportions when echosounders were passive (52%, *n* = 42 groups, 102 individuals) versus active (48%, *n* = 39 groups, 80 individuals). Numbers of groups sighted per day ranged from 0 (7 days) to 10 (1 day), with a median of 1 group/day. In 2013, many more beaked whales were sighted, with a total of 175 groups (*n* = 456 individuals) across 22 days. Of these, 66% were sighted when echosounders were passive (*n* = 114 groups, 309 individuals), while 34% of groups were sighted when echosounders were active (*n* = 61 groups, 147 individuals). Two days in passive mode had extremely high numbers of sightings (*n* = 42 and 47 groups). Both of these days occurred in the Georges Bank region and in low sea state conditions (figure 4). Numbers of groups sighted per day ranged from 0 (8 days) to 47 (1 day), with a median of 2 groups per day. Numbers of groups sighted decreased as sea state increased (table 3).

Visual data from 2011 and 2013 were combined as there were few days between the two surveys when the median sea state was ‘low’ (*n* = 8 for 2011, *n* = 9 for 2013). In the fully saturated additive model, both sea state and region were significant at the 95% level (*p* < 0.001; *p* = 0.04, respectively). None of the explanatory variables showed evidence of collinearity (VIF < 1.1 for all). A model that included an interaction term between echosounder and sea state was also evaluated; this model had a higher AIC value and the interaction term was not significant, therefore the additive model was used for the rest of the analyses (see electronic supplementary material for additional model fit data). The negative binomial GLM with the lowest AIC score from both the backwards stepwise selection and single-term deletion methods was a model with sea state + region covariates (table 5). A model that also included echosounder, and the fully saturated model, each had ΔAIC < 2 as compared to the sea state + region model, but neither showed an improvement in the maximized log-likelihood or residual deviance (table 5). Therefore, the sea state + region model was considered the most parsimonious. Cook's distance (*D*) was used to assess whether observations of potential influence existed in this model. The mean Cook's distance value was *D* = 0.02, and the two days with high numbers of sightings during low sea states had values of *D* = 0.19 and *D* = 0.33. Using criteria that define outliers as *D* > 1, these days were not excluded. A comparison of mean values adjusted to be on the response scale from the best fit model showed nearly a fourfold increase in sightings in low sea states, in both the southern New England and Georges Bank regions (table 6).

**Table 5.** Stepwise AIC selection process for all datasets. The starting model (full model) for both acoustic datasets and visual dataset was a count ∼ echosounder + region + habitat type + sea state + offset(log(trackline distance)) negative binomial. The top three models are shown for each dataset, with the best fit model indicated by an asterisk.

	resid. d.f.	resid. deviance	AIC	ΔAIC
visual 2013 and 2011 data combined				
sea state + region	60	66.91	267.96	0.0*
sea state + region + echosounder	59	67.15	267.97	0.01
sea state + region + echosounder + habitat	58	67.1	269.01	1.05
acoustic BEAK 2013 data				
echosounder + region	30	33.1	117.97	0.0*
echosounder + region + habitat	29	33.2	119.87	1.9
echosounder + region + habitat + sea state	28	33.5	121.78	3.81
acoustic all beaked events 2013 data				
echosounder + region	30	33.5	141.84	0.0*
echosounder + region + habitat	29	33.55	143.83	1.99
echosounder + region + habitat + sea state	28	33.6	145.83	3.99

**Table 6.** Mean sightings and acoustic detections adjusted to the response scale, based on the interaction between sea state and region (visual) and echosounder and region (acoustic), for the best fit models. s.e. indicates standard error. For region, Georges refers to Georges Bank, SNE refers to southern New England.

visual model				acoustic BEAK model			
sea state	region	adjusted mean	s.e.	echosounder	region	adjusted mean	s.e.
low	Georges	8.8	0.24	passive	Georges	9.0	0.33
high	Georges	2.3	0.22	active	Georges	0.4	0.56
low	SNE	4.6	0.26	passive	SNE	2.6	0.37
high	SNE	1.2	0.21	active	SNE	0.1	0.63

**Figure 5.**
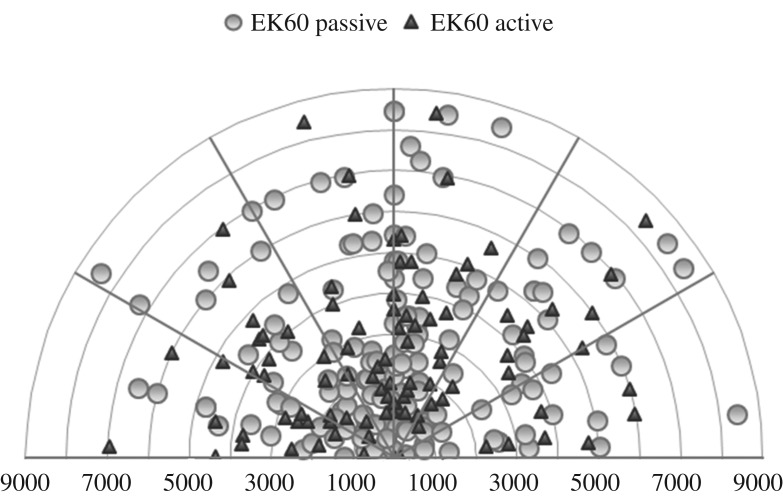
Distribution of radial distances to beaked whale groups sighted ahead and alongside the ship, from 0° to 90° port or starboard. Distances are metres from the ship (centre).

Average radial distances to sightings were 3692 m (±2354) when echosounders were in passive mode, and 3438 m (±1890) when echosounders were active (figure 5). The distributions of radial distances were not significantly different between echosounder states (K-S test, *n* = 256 groups, *D*_mvd_ = 0.08, *p* = 0.83).

**Figure 6.**
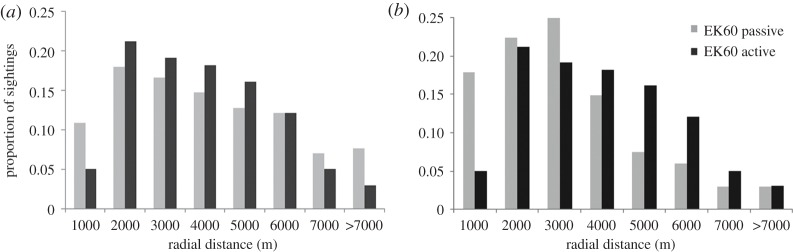
Frequency histogram of radial distances to sightings. (*a*) Proportion of sightings by distance for full dataset; (*b*) proportion of sightings by distance where the two days with extremely high sightings were removed.

We conducted a sensitivity analysis to determine the effect of the two high sighting days on our results. When excluded from the dataset, the best approximating visual model maintained only sea state, with region dropping out. Removing those two days also changed the distribution of sightings (figure 6), such that groups were sighted significantly further from the ship when echosounders were active compared to when they were passive (active mean = 3515 ± 1904 km; passive mean = 2748 ± 1838, K-S test, *n* = 167 groups, *D*_mvd_ = 0.25, *p* = 0.01).

### 3.3. Acoustic detection data

A total of 183 beaked whale events were acoustically detected, including all three categories (BEAK, PRBK, POBK). Of these, 118 were considered definite beaked whale events (BEAK). While we did not subdivide acoustic data by species for this study, the majority of detections had spectral characteristics consistent with Cuvier's beaked whales (*Ziphius cavirostris*; [34]) or with beaked whales of the genus *Mesoplodon* that may be Gervais' (*M. europaeus*; [36]) or True's (*M. mirus*) beaked whales.

The number of beaked whale events per day in all three categories ranged from 0 (17 days) to 28 (1 day). In the overall dataset, as well as the subset of only BEAK events, 96% of detections occurred when echosounders were in passive mode (*n* = 176 and *n* = 114 groups, respectively; see table 4 for BEAK events). Detections per kilometre averaged 0.04 when echosounders were in passive mode, and decreased to less than 0.01 when echosounders were active (table 4).

For the BEAK dataset, in the fully saturated model, both echosounder and region were significant at the 95% level (*p* < 0.001; *p* = 0.01, respectively). None of the explanatory variables showed evidence of collinearity (VIF < 1.3 for all). Negative binomial GLM results from both the backwards stepwise and single-term deletion methods indicated that an additive model with echosounder state and region best explained the data (table 5). In the dataset including BEAK, PRBK and POBK, only echosounder was significant in the fully saturated model (*p* < 0.001). However, the final model using both the backwards stepwise and single-term deletion methods maintained both echosounder and region (table 5). In both datasets, no observations were considered to be significantly influential (Cook's distance less than 0.3 for all; see electronic supplementary material for additional model fit data). A comparison of mean values from the best fit model for the BEAK dataset showed a 20-fold increase in detections when echosounders were in passive mode, regardless of region (table 6).

**Figure 7.**
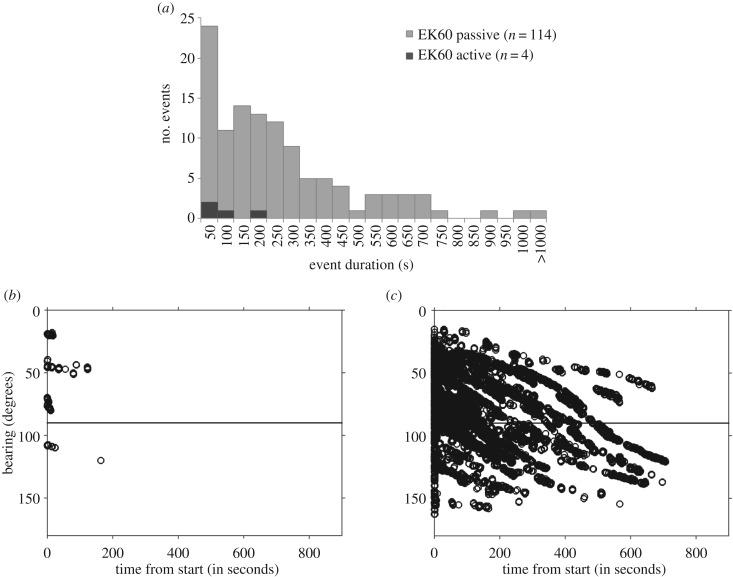
Top panel: (*a*) frequency histogram of the duration of beaked whale acoustic events when echosounders were in passive mode (light grey bars), and when echosounders were active (black bars). Bottom panels: bearings relative to the ship for acoustically tracked beaked whales in the BEAK category, such that 0° is straight ahead of the ship and 180° is directly behind the ship, where (*b*) echosounders were operated in active mode, and (*c*) echosounders were operated in passive mode. Detections of individual echolocation clicks within events are denoted by open circles.

The duration of acoustic encounters and the change in bearing of animals relative to the ship during the encounter were evaluated for BEAK events. Because there were only 4 BEAK events when echosounders were active compared to 114 events when echosounders were passive, statistical comparisons were not feasible. When echosounders were in passive mode, BEAK events ranged in duration from 1 to 1009 s, with a median duration of 172 s (figure 7). Initial bearings of animals relative to the ship ranged from approximately 15–160°, indicating that animals were detected in front of, alongside, and behind the ship. The change in bearing for animals that were tracked as the ship passed by ranged from a few degrees up to 114° (figure 7). In contrast, when echosounders were active, the duration of BEAK events ranged from 10 to 164 s, with a median duration of 49 s. None of these events exceeded a 13° change in bearing.

## 4. Discussion

In our study, beaked whales were significantly less likely to be detected acoustically when our suite of shipboard echosounders were actively transmitting. Only 4 of 118 BEAK events (3%), or 7 out of the 183 combined acoustic events (4%) took place when the EK60s were transmitting. When beaked whales were detected during active echosounder use, they were detected for less time (median duration of 49 s, compared to 172 s when the EK60 was in passive mode), and were consequently tracked over a smaller range of bearings relative to the ship. In addition to echosounders, region also had an impact on the probability of acoustically detecting beaked whales, with more groups detected in the region along and offshore Georges Bank. Sea state did not affect the probability of acoustically detecting beaked whales.

In contrast to the acoustic data, sea state was the primary driver for sighting beaked whales. This was not surprising, as prior research has demonstrated a reduction in the sightability of these species as sea state conditions worsen [39], with a decrease in encounter rate of 10-fold or more as Beaufort sea states changes from 0 to 5. In our study, 68% of groups were sighted in ‘low’ Beaufort conditions, even though those low sea state days comprised only 26% of the survey effort. Echosounder state was not included in the best fit model, and the distribution of radial distances to sightings was not different between echosounder states.

However, these results should be treated with caution. Our study included relatively few days with low sea states when echosounders were in passive mode (6/63 days), and in two of those days, almost three times the number of groups were sighted as compared to the highest number of groups sighted when echosounders were active (42 & 47 groups versus 17 groups). Our sensitivity analysis indicated that removing those two days changed the distribution of sightings, resulting in a significant difference in radial distances, such that animals are sighted further from the ship when echosounders are active. However, there was nothing anomalous about these days except for the high numbers of sightings, and examination of the data revealed no reason for excluding them. Therefore, it is difficult to determine whether they are outliers or whether they instead reflect a real increase in sightability of beaked whales when sea states are low in the Georges Bank region, and our analysis may not be sensitive enough to fully evaluate the relationship between beaked whale sightings and echosounder use. The interpretation of the visual data is complex, and further work is needed to better understand the influence of echosounders on sighting distributions and rates.

Nevertheless, the reduction in both the number of acoustic events and the decrease in duration of BEAK events (correlating to the smaller degrees in bearing change) when echosounders are operating indicate that beaked whales are both detecting and responding behaviourally to the presence of shipboard echosounders. While the mechanism of this response is not known, DeRuiter *et al.* [43] found that beaked whales exposed to mid-frequency active sonar exhibited a silent flight response, in which they moved away from the study area without echolocating. In a study in which pilot whales equipped with digital recording tags were exposed to scientific echosounders [18], individuals exhibited an increased variance in heading when echosounders were active, suggesting an increase in vigilance. In the current study, beaked whales may be responding to the presence of shipboard echosounders in multiple ways: they may move out of our detection range, or initiate directed movement away from the ship, or they may remain in the area but temporarily suspend foraging activity.

Previous papers have suggested that shipboard echosounders are unlikely to cause significant rates of injury to cetaceans, due to their relatively narrow beam widths and high absorption coefficients for the higher-frequency sonars [44,45]. However, it is important to distinguish between injury and behavioural impacts. While it may be the case that relatively few animals would be expected to be found directly within the beam of the echosounder and therefore exposed to potentially harmful levels of acoustic signals, it is also the case that these signals are detectable well beyond the direct cone of impact beneath the ship. *In situ* data from bottom-mounted recorders found that the EK60 echosounders used by the R/V *Henry Bigelow* can be detected at 800 m depth out to a distance of at least 1.3 km (unpublished data). Acoustic detection range is influenced by multiple factors; in addition to the direct path of the primary lobe of the transmitted signal, some energy is also transmitted through side lobes, which varies between transducers and can spread acoustic energy horizontally. Also, reflection and scattering off the seafloor are an important component of sound propagation and redirect sound energy at a variety of angles back up into the water column. Lurton [45] modelled the sound fields radiated by three different types of multibeam echosounders; his results for one 12 kHz MBES suggest that the 160 dB isopleth for peak amplitude extends out to nearly 4 km, while SEL measurements were over 140 dB at 2–3 km. While these levels may not be high enough to warrant concern from injurious impact, they are certainly detectable above background noise levels and could elicit changes in behaviour by sensitive species.

These results have implications for management and mitigation of human activities, as well as for conservation. On the management front, NOAA is responsible for estimating stock size and structure for all marine mammal populations that occur within US waters. There has been an increasing push in recent years to incorporate passive acoustic methodologies into stock assessment surveys, particularly to assist in detection of deep-diving species such as beaked whales and sperm whales, which are more difficult to detect visually. However, the use of shipboard echosounders to collect prey field data and conduct bottom-mapping activities is also widespread during scientific surveys. Given that the use of shipboard echosounders has a negative effect on the acoustic detection rates of beaked whales, any passive acoustic data collected while echosounders are operating could be compromised for those species. Our current sample is not large enough to determine if there are particular echosounders that are more likely to induce a response; however, we would predict that echosounders whose frequencies fall directly within the range of echolocation frequencies for beaked whales might have the greatest effect on behavioural response.

With respect to mitigation of human activities, offshore oil and gas exploration continues to expand worldwide, with the potential to directly impact areas of beaked whale habitat along the shelf break and into deeper waters. Because seismic survey technologies often generate low-frequency acoustic signals through the use of airguns, it is commonly assumed that they have little impact on odontocetes species, who use higher frequencies for their whistles and echolocation clicks. However, many of these vessels also operate numerous echosounders, the effects of which are often overlooked when quantifying the environmental impact of those activities. While the placement of visual observers on the vessels is standard practice, passive acoustic monitoring is also becoming common as a means of detecting animals that may be within the mitigation zone. Our work indicates that acoustic detection of beaked whales, and subsequent mitigation, may be compromised for vessels that are simultaneously operating echosounders.

Finally, previous studies have demonstrated long-term consequences that may result from frequent short-term disruption, for example, when evaluating the impacts of whale-watching [46]. While individual animals may tolerate a certain level of disturbance, this disturbance may have energetic consequences, physiological (i.e. stress) consequences, and may eventually lead to habitat displacement or abandonment [47]. The level of acoustic disturbance for the beaked whale populations in our study area is unknown. While our surveys are infrequent and transient, other research, fishing and industrial vessels are also operating in offshore waters. At one site along the shelf break within our study area, shipboard echosounders were detected on a bottom-mounted recorder on at least 44 separate days in a six month period (unpublished data). Some populations of beaked whales are known to have high site fidelity (e.g. [48]), which means that the same individuals could be repeatedly exposed to disturbance if anthropogenic activities are frequent in particular areas. This could be cause for concern for sensitive populations.

In summary, our study demonstrated a marked acoustic response of beaked whales to the use of shipboard echosounders. It is important to keep in mind that our study used only SBES, which have relatively narrow directivity and therefore might have less impact when compared to MBES and omnidirectional sonars. These systems are frequently used in a variety of scientific and industrial applications, and we would predict that the transmission ranges and therefore their acoustic impact would be greater. Considerations of the effects of echosounder use on cetaceans should be taken into account when evaluating the environmental impact of vessel-based activities, when planning for ecosystem monitoring activities using active acoustics, and when conducting vessel-based surveys to determine the abundance of beaked whales.

## Supplementary Material

RSOS_17049_Visual_data_code_201711

## Supplementary Material

visual_groups_w_region

## Supplementary Material

acoustic_ALL_w_region

## Supplementary Material

acoustic_BEAK_w_region

## Supplementary Material

RSOS_170940_Acoustic_data_code_201711

## Data accessibility

Visual and acoustic detection data that were used as model inputs, as well as the R code associated with the statistical analyses, are available as electronic supplementary material.
