# Supplementary material for: Beaked whales demonstrate a marked acoustic response to the use of shipboard echosounders
Source: R Soc Open Sci. 2017 Dec 13;4(12):170940. doi: 10.1098/rsos.170940 (PMC5750003; doi:10.1098/rsos.170940)
Supplement: RSOS_17049_Visual_data_code_201711 [file rsos170940supp1.html]

RSOS 170940 - Testing the effects of echosounders on beaked whale detections- Visual dataset


# RSOS 170940 - Testing the effects of echosounders on beaked whale detections- Visual dataset

#### *November 3, 2017*

```
#empty workspace
rm(list=ls())
#load data
visualData<- read.table("visual_data_w_region.txt", header= T)

#set echosounder & seastate as factors
visualData$Echosounder<-as.factor(visualData$Echosounder)
visualData$SeaState<-as.factor(visualData$SeaState)
str(visualData)
```

```
## 'data.frame':    63 obs. of  9 variables:
##  $ Day              : int  1 2 3 4 5 6 7 8 9 10 ...
##  $ Date             : Factor w/ 63 levels "6/10/2011","6/11/2011",..: 14 15 16 17 18 19 1 2 3 4 ...
##  $ nTotal           : int  1 0 7 1 0 6 0 0 0 3 ...
##  $ Echosounder      : Factor w/ 2 levels "0","1": 2 1 2 1 2 1 2 1 2 1 ...
##  $ SeaStateMed      : num  4 3 2.8 4.7 4.4 3 3.95 4.4 4.8 3.4 ...
##  $ SeaState         : Factor w/ 2 levels "high","low": 1 1 2 1 1 1 1 1 1 1 ...
##  $ HabitatType      : Factor w/ 2 levels "Abyssal","Slope": 2 2 2 2 1 2 1 2 2 2 ...
##  $ Region           : Factor w/ 2 levels "Georges","SNE": 2 2 2 2 2 1 1 1 1 1 ...
##  $ TracklineDist.nmi: num  83.8 98.4 90.5 50.1 28.4 ...
```

### Overdispersion

Checking to see if there’s overdispersion in the data by using a quasipoisson to estimate phi.

```
require(MASS)
```

```
## Loading required package: MASS
```

```
visualquasip<- glm(nTotal~Echosounder+SeaState+HabitatType+Region,data=visualData,
                   family= "quasipoisson",offset=log(TracklineDist.nmi))
summary(visualquasip)
```

```
## 
## Call:
## glm(formula = nTotal ~ Echosounder + SeaState + HabitatType + 
##     Region, family = "quasipoisson", data = visualData, offset = log(TracklineDist.nmi))
## 
## Deviance Residuals: 
##     Min       1Q   Median       3Q      Max  
## -3.9631  -1.3382  -0.4073   0.5540   4.8330  
## 
## Coefficients:
##                  Estimate Std. Error t value Pr(>|t|)    
## (Intercept)       -2.8330     0.2663 -10.639 2.97e-15 ***
## Echosounder1      -0.5314     0.2506  -2.121   0.0382 *  
## SeaStatelow        1.3945     0.2656   5.251 2.25e-06 ***
## HabitatTypeSlope  -0.3906     0.2581  -1.513   0.1356    
## RegionSNE         -0.6842     0.2602  -2.629   0.0109 *  
## ---
## Signif. codes:  0 '***' 0.001 '**' 0.01 '*' 0.05 '.' 0.1 ' ' 1
## 
## (Dispersion parameter for quasipoisson family taken to be 3.600922)
## 
##     Null deviance: 406.81  on 62  degrees of freedom
## Residual deviance: 206.82  on 58  degrees of freedom
## AIC: NA
## 
## Number of Fisher Scoring iterations: 5
```

The data are overdispersed, (phi= 3.6009223), using a negative binomial model to address the overdispersion.

```
visualnegB<-glm.nb(nTotal~Echosounder+SeaState+HabitatType+Region+offset(log(TracklineDist.nmi)), data=visualData,link= "log")
summary(visualnegB)
```

```
## 
## Call:
## glm.nb(formula = nTotal ~ Echosounder + SeaState + HabitatType + 
##     Region + offset(log(TracklineDist.nmi)), data = visualData, 
##     link = "log", init.theta = 1.726319187)
## 
## Deviance Residuals: 
##     Min       1Q   Median       3Q      Max  
## -2.1403  -1.1518  -0.2135   0.3406   1.6680  
## 
## Coefficients:
##                  Estimate Std. Error z value Pr(>|z|)    
## (Intercept)       -3.0059     0.2693 -11.162  < 2e-16 ***
## Echosounder1      -0.3688     0.2659  -1.387   0.1655    
## SeaStatelow        1.2916     0.2761   4.678  2.9e-06 ***
## HabitatTypeSlope  -0.2661     0.2621  -1.015   0.3100    
## RegionSNE         -0.5238     0.2594  -2.019   0.0435 *  
## ---
## Signif. codes:  0 '***' 0.001 '**' 0.01 '*' 0.05 '.' 0.1 ' ' 1
## 
## (Dispersion parameter for Negative Binomial(1.7263) family taken to be 1)
## 
##     Null deviance: 111.351  on 62  degrees of freedom
## Residual deviance:  67.074  on 58  degrees of freedom
## AIC: 271.01
## 
## Number of Fisher Scoring iterations: 1
## 
## 
##               Theta:  1.726 
##           Std. Err.:  0.535 
## 
##  2 x log-likelihood:  -259.007
```

The data are no longer as overdispersed (theta= 1.7263192).

### Model selection

Test whether an additive model or an interaction between echosounder and sea state is more parsimonious.

```
require(car)
```

```
## Loading required package: car
```

```
visualnegBInt<- glm.nb(nTotal~Echosounder*SeaState+HabitatType+Region+offset(log(TracklineDist.nmi)), data=visualData,link= "log")
summary(visualnegBInt)
```

```
## 
## Call:
## glm.nb(formula = nTotal ~ Echosounder * SeaState + HabitatType + 
##     Region + offset(log(TracklineDist.nmi)), data = visualData, 
##     link = "log", init.theta = 1.771067737)
## 
## Deviance Residuals: 
##     Min       1Q   Median       3Q      Max  
## -2.1068  -1.2123  -0.3250   0.4367   1.5049  
## 
## Coefficients:
##                          Estimate Std. Error z value Pr(>|z|)    
## (Intercept)               -3.1250     0.2875 -10.869  < 2e-16 ***
## Echosounder1              -0.1926     0.3322  -0.580   0.5620    
## SeaStatelow                1.5608     0.3961   3.941 8.13e-05 ***
## HabitatTypeSlope          -0.2237     0.2606  -0.859   0.3906    
## RegionSNE                 -0.4872     0.2589  -1.882   0.0598 .  
## Echosounder1:SeaStatelow  -0.4814     0.5361  -0.898   0.3692    
## ---
## Signif. codes:  0 '***' 0.001 '**' 0.01 '*' 0.05 '.' 0.1 ' ' 1
## 
## (Dispersion parameter for Negative Binomial(1.7711) family taken to be 1)
## 
##     Null deviance: 112.970  on 62  degrees of freedom
## Residual deviance:  67.091  on 57  degrees of freedom
## AIC: 272.23
## 
## Number of Fisher Scoring iterations: 1
## 
## 
##               Theta:  1.771 
##           Std. Err.:  0.555 
## 
##  2 x log-likelihood:  -258.227
```

```
AIC(visualnegB)
```

```
## [1] 271.0072
```

```
AIC(visualnegBInt)
```

```
## [1] 272.2271
```

```
Anova(visualnegBInt)
```

```
## Analysis of Deviance Table (Type II tests)
## 
## Response: nTotal
##                      LR Chisq Df Pr(>Chisq)    
## Echosounder            1.9128  1    0.16665    
## SeaState              22.6284  1  1.966e-06 ***
## HabitatType            0.6821  1    0.40886    
## Region                 3.2964  1    0.06943 .  
## Echosounder:SeaState   0.7868  1    0.37506    
## ---
## Signif. codes:  0 '***' 0.001 '**' 0.01 '*' 0.05 '.' 0.1 ' ' 1
```

Using both AIC and Anova tests, we see that the additive model is more parsimonious (slightly lower AIC value and the p-value is not significant for the interaction in the Anova), so we will be conducting the rest of the model assessment with the additive model.

Next, perform model selection to see which covariates to keep in the model.

```
visualnegBStep<- stepAIC(visualnegB) #backwards model selection, echosounder and sea state left in the model
```

```
## Start:  AIC=269.01
## nTotal ~ Echosounder + SeaState + HabitatType + Region + offset(log(TracklineDist.nmi))
## 
##               Df    AIC
## - HabitatType  1 267.97
## - Echosounder  1 268.84
## <none>           269.01
## - Region       1 270.57
## - SeaState     1 284.78
## 
## Step:  AIC=267.97
## nTotal ~ Echosounder + SeaState + Region + offset(log(TracklineDist.nmi))
## 
##               Df    AIC
## - Echosounder  1 267.96
## <none>           267.97
## - Region       1 269.58
## - SeaState     1 287.77
## 
## Step:  AIC=267.96
## nTotal ~ SeaState + Region + offset(log(TracklineDist.nmi))
## 
##            Df    AIC
## <none>        267.96
## - Region    1 271.26
## - SeaState  1 285.99
```

```
visualnegBStep$anova #step-wise model selection
```

```
## Stepwise Model Path 
## Analysis of Deviance Table
## 
## Initial Model:
## nTotal ~ Echosounder + SeaState + HabitatType + Region + offset(log(TracklineDist.nmi))
## 
## Final Model:
## nTotal ~ SeaState + Region + offset(log(TracklineDist.nmi))
## 
## 
##            Step Df   Deviance Resid. Df Resid. Dev      AIC
## 1                                    58   67.07417 269.0072
## 2 - HabitatType  1 0.08069162        59   67.15486 267.9690
## 3 - Echosounder  1 0.24525363        60   66.90960 267.9631
```

```
summary(visualnegBStep)
```

```
## 
## Call:
## glm.nb(formula = nTotal ~ SeaState + Region + offset(log(TracklineDist.nmi)), 
##     data = visualData, init.theta = 1.565634368, link = "log")
## 
## Deviance Residuals: 
##     Min       1Q   Median       3Q      Max  
## -2.2582  -1.1134  -0.3075   0.3225   1.9856  
## 
## Coefficients:
##             Estimate Std. Error z value Pr(>|z|)    
## (Intercept)  -3.2604     0.2187 -14.905  < 2e-16 ***
## SeaStatelow   1.3211     0.2721   4.854 1.21e-06 ***
## RegionSNE    -0.6396     0.2651  -2.413   0.0158 *  
## ---
## Signif. codes:  0 '***' 0.001 '**' 0.01 '*' 0.05 '.' 0.1 ' ' 1
## 
## (Dispersion parameter for Negative Binomial(1.5656) family taken to be 1)
## 
##     Null deviance: 105.31  on 62  degrees of freedom
## Residual deviance:  66.91  on 60  degrees of freedom
## AIC: 269.96
## 
## Number of Fisher Scoring iterations: 1
## 
## 
##               Theta:  1.566 
##           Std. Err.:  0.464 
## 
##  2 x log-likelihood:  -261.963
```

```
#dropterm
visdrop<- dropterm(visualnegB, test= "Chisq") 
visdrop
```

```
## Single term deletions
## 
## Model:
## nTotal ~ Echosounder + SeaState + HabitatType + Region + offset(log(TracklineDist.nmi))
##             Df    AIC     LRT   Pr(Chi)    
## <none>         269.01                      
## Echosounder  1 268.84  1.8346   0.17558    
## SeaState     1 284.78 17.7683 2.495e-05 ***
## HabitatType  1 267.97  0.9618   0.32673    
## Region       1 270.57  3.5635   0.05906 .  
## ---
## Signif. codes:  0 '***' 0.001 '**' 0.01 '*' 0.05 '.' 0.1 ' ' 1
```

#### Testing the other model assumptions for the reduced echosounder + sea state model.

### Checking for collinearity

```
vif(visualnegBStep)
```

```
## SeaState   Region 
## 1.004317 1.004317
```

Both covariates of echosounder and sea state have variance inflation factors (VIFs) ~1 therefore there is no collinearity.

### Linearity on the function’s link scale (Echosounder + sea state)

```
par(mfrow=c(1,2))
plot(visualData$nTotal,fitted(visualnegBStep), xlab= "Observed values", ylab= "Fitted values", main= "Linearity on the log link scale")
abline(0,1)
#scaling Pearson residuals to take into account overdispersion
phi<- summary(visualnegBStep)$theta
fits<- fitted(visualnegBStep, type= "response")
scaledresid<- (visualData$nTotal-fits)/sqrt(phi*fits)
plot(fits,scaledresid, xlab= "Fitted values", ylab= "Scaled residuals", main= "Model fit-residuals")
abline(h=0)
```

### Influence

```
par(mfrow=c(1,1))
plot(cooks.distance(visualnegBStep),main= "Influence of observations") #Cook's distance < 1, pass
```

There does not appear to be any gross influence by any of the observations, though two days may be somewhat influential based on their higher Cook’s Distance values.

### Testing for independence

```
acf(visualnegBStep$residuals, main= "Independence of model residuals") #within CI, pass
```

There is no violation of the independence assumption.

### Confidence intervals for covariates

```
confint(visualnegBStep)
```

```
## Waiting for profiling to be done...
```

```
##                  2.5 %     97.5 %
## (Intercept) -3.6949578 -2.8156952
## SeaStatelow  0.7951694  1.8689000
## RegionSNE   -1.1635880 -0.1158713
```

### Visualizing the data by sea state and region

```
require(ggplot2)
```

```
## Loading required package: ggplot2
```

```
require(phia)
```

```
## Loading required package: phia
```

```
require(ggfortify)
```

```
## Loading required package: ggfortify
```

```
## Warning: namespace 'DBI' is not available and has been replaced
## by .GlobalEnv when processing object 'silent'

## Warning: namespace 'DBI' is not available and has been replaced
## by .GlobalEnv when processing object 'silent'
```

```
p<-ggplot(visualData, aes(SeaState,nTotal,colour=Region))+geom_count()
p
```

### Test of contrasts: Understanding the relationship between echosounder and sea state covariates

```
visualnegBMeans<-interactionMeans(visualnegBStep)
visualnegBMeans
```

```
##   SeaState  Region adjusted mean SE of link
## 1     high Georges      2.338079  0.2187500
## 2      low Georges      8.761805  0.2447155
## 3     high     SNE      1.233376  0.2107952
## 4      low     SNE      4.622001  0.2567642
```

```
plot(visualnegBMeans)
```
