# Supplementary material for: Beaked whales demonstrate a marked acoustic response to the use of shipboard echosounders
Source: R Soc Open Sci. 2017 Dec 13;4(12):170940. doi: 10.1098/rsos.170940 (PMC5750003; doi:10.1098/rsos.170940)
Supplement: RSOS_170940_Acoustic_data_code_201711 [file rsos170940supp5.html]

RSOS 170940 - Testing the effects of echosounders on beaked whales - Acoustic dataset


# RSOS 170940 - Testing the effects of echosounders on beaked whales - Acoustic dataset

```
# create empty workspace
rm(list=ls())

#load data
acousticBEAK<- read.table("acoustic_BEAK_w_region.txt", header= T)
acousticALL<- read.table("acoustic_ALL_w_region.txt", header= T)

#set echosounder & seastate as factors
acousticBEAK$Echosounder<-as.factor(acousticBEAK$Echosounder)
acousticBEAK$SeaState<-as.factor(acousticBEAK$SeaState)
```

## Acoustic BEAK dataset

### Overdispersion

Checking to see if there’s overdispersion in the data by using a quasipoisson to estimate phi.

```
BEAKquasip<- glm(nTotal~Echosounder+SeaState+HabitatType+Region,data=acousticBEAK,
                   family= "quasipoisson",offset=log(TracklineDist.nmi))
summary(BEAKquasip)
```

```
## 
## Call:
## glm(formula = nTotal ~ Echosounder + SeaState + HabitatType + 
##     Region, family = "quasipoisson", data = acousticBEAK, offset = log(TracklineDist.nmi))
## 
## Deviance Residuals: 
##     Min       1Q   Median       3Q      Max  
## -2.6332  -1.1298  -0.4523   0.5716   3.2026  
## 
## Coefficients:
##                  Estimate Std. Error t value Pr(>|t|)    
## (Intercept)       -1.7637     0.2811  -6.274 8.79e-07 ***
## Echosounder1      -3.1376     0.9831  -3.192  0.00348 ** 
## SeaStatelow       -0.4768     0.4857  -0.982  0.33463    
## HabitatTypeSlope  -0.2267     0.3896  -0.582  0.56528    
## RegionSNE         -1.6025     0.4826  -3.321  0.00251 ** 
## ---
## Signif. codes:  0 '***' 0.001 '**' 0.01 '*' 0.05 '.' 0.1 ' ' 1
## 
## (Dispersion parameter for quasipoisson family taken to be 3.644569)
## 
##     Null deviance: 262.580  on 32  degrees of freedom
## Residual deviance:  74.051  on 28  degrees of freedom
## AIC: NA
## 
## Number of Fisher Scoring iterations: 6
```

The data are overdispersed, (phi= 3.6445691), using a negative binomial model to address the overdispersion.

```
require(MASS)
BEAKnegB<-glm.nb(nTotal~Echosounder+SeaState+HabitatType+Region+offset(log(TracklineDist.nmi)), data=acousticBEAK,link= "log")
summary(BEAKnegB)
```

```
## 
## Call:
## glm.nb(formula = nTotal ~ Echosounder + SeaState + HabitatType + 
##     Region + offset(log(TracklineDist.nmi)), data = acousticBEAK, 
##     link = "log", init.theta = 1.247592477)
## 
## Deviance Residuals: 
##     Min       1Q   Median       3Q      Max  
## -1.8332  -0.9337  -0.5094   0.4127   1.7915  
## 
## Coefficients:
##                  Estimate Std. Error z value Pr(>|z|)    
## (Intercept)       -1.9882     0.4314  -4.609 4.04e-06 ***
## Echosounder1      -2.9750     0.6370  -4.670 3.01e-06 ***
## SeaStatelow       -0.2059     0.6532  -0.315   0.7525    
## HabitatTypeSlope  -0.1980     0.4864  -0.407   0.6839    
## RegionSNE         -1.2632     0.4933  -2.561   0.0104 *  
## ---
## Signif. codes:  0 '***' 0.001 '**' 0.01 '*' 0.05 '.' 0.1 ' ' 1
## 
## (Dispersion parameter for Negative Binomial(1.2476) family taken to be 1)
## 
##     Null deviance: 81.442  on 32  degrees of freedom
## Residual deviance: 33.522  on 28  degrees of freedom
## AIC: 123.78
## 
## Number of Fisher Scoring iterations: 1
## 
## 
##               Theta:  1.248 
##           Std. Err.:  0.692 
## 
##  2 x log-likelihood:  -111.778
```

The data are less overdispersed (theta 1.2475925).

### Model selection

Next, see which covariates to keep in the model.

```
BEAKnegBStep<- stepAIC(BEAKnegB) #backwards model selection, region and echosounder left in the model
```

```
## Start:  AIC=121.78
## nTotal ~ Echosounder + SeaState + HabitatType + Region + offset(log(TracklineDist.nmi))
## 
##               Df    AIC
## - SeaState     1 119.87
## - HabitatType  1 119.94
## <none>           121.78
## - Region       1 124.85
## - Echosounder  1 134.70
## 
## Step:  AIC=119.87
## nTotal ~ Echosounder + HabitatType + Region + offset(log(TracklineDist.nmi))
## 
##               Df    AIC
## - HabitatType  1 117.97
## <none>           119.87
## - Region       1 122.92
## - Echosounder  1 134.91
## 
## Step:  AIC=117.97
## nTotal ~ Echosounder + Region + offset(log(TracklineDist.nmi))
## 
##               Df    AIC
## <none>           117.97
## - Region       1 121.02
## - Echosounder  1 133.41
```

```
BEAKnegBStep$anova #step-wise model selection
```

```
## Stepwise Model Path 
## Analysis of Deviance Table
## 
## Initial Model:
## nTotal ~ Echosounder + SeaState + HabitatType + Region + offset(log(TracklineDist.nmi))
## 
## Final Model:
## nTotal ~ Echosounder + Region + offset(log(TracklineDist.nmi))
## 
## 
##            Step Df  Deviance Resid. Df Resid. Dev      AIC
## 1                                   28   33.52162 121.7784
## 2    - SeaState  1 0.3364470        29   33.18518 119.8658
## 3 - HabitatType  1 0.1087067        30   33.07647 117.9709
```

```
summary(BEAKnegBStep)
```

```
## 
## Call:
## glm.nb(formula = nTotal ~ Echosounder + Region + offset(log(TracklineDist.nmi)), 
##     data = acousticBEAK, init.theta = 1.187692015, link = "log")
## 
## Deviance Residuals: 
##     Min       1Q   Median       3Q      Max  
## -1.8426  -0.9962  -0.5294   0.4370   1.6921  
## 
## Coefficients:
##              Estimate Std. Error z value Pr(>|z|)    
## (Intercept)   -2.1127     0.3319  -6.365 1.96e-10 ***
## Echosounder1  -3.0715     0.6010  -5.110 3.22e-07 ***
## RegionSNE     -1.2422     0.4752  -2.614  0.00895 ** 
## ---
## Signif. codes:  0 '***' 0.001 '**' 0.01 '*' 0.05 '.' 0.1 ' ' 1
## 
## (Dispersion parameter for Negative Binomial(1.1877) family taken to be 1)
## 
##     Null deviance: 79.227  on 32  degrees of freedom
## Residual deviance: 33.076  on 30  degrees of freedom
## AIC: 119.97
## 
## Number of Fisher Scoring iterations: 1
## 
## 
##               Theta:  1.188 
##           Std. Err.:  0.645 
## 
##  2 x log-likelihood:  -111.971
```

```
#dropterm
BEAKdrop<- dropterm(BEAKnegB, test= "Chisq") 
BEAKdrop
```

```
## Single term deletions
## 
## Model:
## nTotal ~ Echosounder + SeaState + HabitatType + Region + offset(log(TracklineDist.nmi))
##             Df    AIC     LRT   Pr(Chi)    
## <none>         121.78                      
## Echosounder  1 134.70 14.9178 0.0001123 ***
## SeaState     1 119.87  0.0874 0.7675238    
## HabitatType  1 119.94  0.1591 0.6900098    
## Region       1 124.85  5.0715 0.0243224 *  
## ---
## Signif. codes:  0 '***' 0.001 '**' 0.01 '*' 0.05 '.' 0.1 ' ' 1
```

Both Region and Echosounder are significant and remain in the model.

Testing other model assumptions.

### Variance inflation factors (VIFs) for collinearity

Checking to see if any covariates are collinear in the reduced model.

```
require(car)
vif(BEAKnegBStep)
```

```
## Echosounder      Region 
##    1.010081    1.010081
```

All VIFs are ~1 which indicates no collinearity of the covariates.

### Linearity on the function’s link scale

```
par(mfrow=c(1,2))
plot(acousticBEAK$nTotal,fitted(BEAKnegBStep), xlab= "Observed values", ylab= "Fitted values", main= "Linearity on the log link scale")
abline(0,1)
#scaling Pearson residuals to take into account overdispersion
phi<- summary(BEAKnegBStep)$theta
fits<- fitted(BEAKnegBStep, type= "response")
scaledresid<- (acousticBEAK$nTotal-fits)/sqrt(phi*fits)
plot(fits,scaledresid, xlab= "Fitted values", ylab= "Scaled residuals", main= "Model fit-residuals")
abline(h=0)
```

The data mostly follow a linear relationship on the function’s log-link scale. Testing the other model assumptions.

### Influence

```
par(mfrow=c(1,1))
plot(cooks.distance(BEAKnegBStep),main= "Influence of observations") #Cook's distance < 1, pass
```

There does not appear to be any gross influence by any of the observations.

### Testing for independence

```
acf(BEAKnegBStep$residuals, main= "Independence of model residuals") #within CI, pass
```

There is no violation of the independence assumption.

```
### Confidence intervals for covariates
```

```
confint(BEAKnegBStep)
```

```
## Waiting for profiling to be done...
```

```
##                  2.5 %     97.5 %
## (Intercept)  -2.694083 -1.4314740
## Echosounder1 -4.438498 -1.9138965
## RegionSNE    -2.201185 -0.2930353
```

### Test of contrasts: Understanding the relationship between echosounder and region covariates

```
require(ggplot2)
require(phia)
require(ggfortify)

BEAKnegBMeans<-interactionMeans(BEAKnegBStep)
BEAKnegBMeans
```

```
##   Echosounder  Region adjusted mean SE of link
## 1           0 Georges     8.9982673  0.3319488
## 2           1 Georges     0.4170945  0.5593899
## 3           0     SNE     2.5982110  0.3713724
## 4           1     SNE     0.1204342  0.6306476
```

```
plot(BEAKnegBMeans)
```

---

## Acoustic ALL dataset

### Overdispersion

Checking to see if there’s overdispersion in the data by using a quasipoisson to estimate phi.

```
ALLquasip<- glm(acousticALL$nTotal~as.factor(acousticALL$Echosounder)+as.factor(acousticALL$Region)+as.factor(acousticALL$HabitatType)+as.factor(acousticALL$SeaState), family= "quasipoisson", offset= log(acousticALL$TracklineDist.nmi))
summary(ALLquasip)
```

```
## 
## Call:
## glm(formula = acousticALL$nTotal ~ as.factor(acousticALL$Echosounder) + 
##     as.factor(acousticALL$Region) + as.factor(acousticALL$HabitatType) + 
##     as.factor(acousticALL$SeaState), family = "quasipoisson", 
##     offset = log(acousticALL$TracklineDist.nmi))
## 
## Deviance Residuals: 
##     Min       1Q   Median       3Q      Max  
## -3.7730  -1.5012  -0.6061   0.7119   4.8979  
## 
## Coefficients:
##                                         Estimate Std. Error t value
## (Intercept)                              -1.4432     0.2982  -4.840
## as.factor(acousticALL$Echosounder)1      -3.0494     0.9489  -3.214
## as.factor(acousticALL$Region)SNE         -1.2788     0.4463  -2.866
## as.factor(acousticALL$HabitatType)Slope  -0.1133     0.3940  -0.288
## as.factor(acousticALL$SeaState)low       -0.4534     0.5150  -0.880
##                                         Pr(>|t|)    
## (Intercept)                             4.29e-05 ***
## as.factor(acousticALL$Echosounder)1      0.00329 ** 
## as.factor(acousticALL$Region)SNE         0.00781 ** 
## as.factor(acousticALL$HabitatType)Slope  0.77583    
## as.factor(acousticALL$SeaState)low       0.38616    
## ---
## Signif. codes:  0 '***' 0.001 '**' 0.01 '*' 0.05 '.' 0.1 ' ' 1
## 
## (Dispersion parameter for quasipoisson family taken to be 5.882929)
## 
##     Null deviance: 389.84  on 32  degrees of freedom
## Residual deviance: 130.96  on 28  degrees of freedom
## AIC: NA
## 
## Number of Fisher Scoring iterations: 6
```

The data are overdispersed, (phi= 5.8829289), using a negative binomial model to address the overdispersion.

```
ALLnegB<- glm.nb(acousticALL$nTotal~as.factor(acousticALL$Echosounder)+as.factor(acousticALL$Region)+as.factor(acousticALL$HabitatType)+as.factor(acousticALL$SeaState)+offset(log(acousticALL$TracklineDist.nmi)), link= "log")
summary(ALLnegB)
```

```
## 
## Call:
## glm.nb(formula = acousticALL$nTotal ~ as.factor(acousticALL$Echosounder) + 
##     as.factor(acousticALL$Region) + as.factor(acousticALL$HabitatType) + 
##     as.factor(acousticALL$SeaState) + offset(log(acousticALL$TracklineDist.nmi)), 
##     link = "log", init.theta = 0.7799373108)
## 
## Deviance Residuals: 
##     Min       1Q   Median       3Q      Max  
## -1.9381  -0.9956  -0.6422   0.3756   1.8864  
## 
## Coefficients:
##                                         Estimate Std. Error z value
## (Intercept)                             -1.77177    0.49256  -3.597
## as.factor(acousticALL$Echosounder)1     -2.94602    0.60785  -4.847
## as.factor(acousticALL$Region)SNE        -0.80009    0.52405  -1.527
## as.factor(acousticALL$HabitatType)Slope -0.04529    0.52505  -0.086
## as.factor(acousticALL$SeaState)low       0.05039    0.68704   0.073
##                                         Pr(>|z|)    
## (Intercept)                             0.000322 ***
## as.factor(acousticALL$Echosounder)1     1.26e-06 ***
## as.factor(acousticALL$Region)SNE        0.126830    
## as.factor(acousticALL$HabitatType)Slope 0.931265    
## as.factor(acousticALL$SeaState)low      0.941530    
## ---
## Signif. codes:  0 '***' 0.001 '**' 0.01 '*' 0.05 '.' 0.1 ' ' 1
## 
## (Dispersion parameter for Negative Binomial(0.7799) family taken to be 1)
## 
##     Null deviance: 68.515  on 32  degrees of freedom
## Residual deviance: 33.546  on 28  degrees of freedom
## AIC: 147.83
## 
## Number of Fisher Scoring iterations: 1
## 
## 
##               Theta:  0.780 
##           Std. Err.:  0.321 
## 
##  2 x log-likelihood:  -135.829
```

The data are no longer overdispersed (theta= 0.7799373).

### Model selection

Next, see which covariates to keep in the model.

```
ALLnegBStep<- stepAIC(ALLnegB) #backwards model selection, month and echosounder left in the model
```

```
## Start:  AIC=145.83
## acousticALL$nTotal ~ as.factor(acousticALL$Echosounder) + as.factor(acousticALL$Region) + 
##     as.factor(acousticALL$HabitatType) + as.factor(acousticALL$SeaState) + 
##     offset(log(acousticALL$TracklineDist.nmi))
## 
##                                      Df    AIC
## - as.factor(acousticALL$SeaState)     1 143.83
## - as.factor(acousticALL$HabitatType)  1 143.84
## <none>                                  145.83
## - as.factor(acousticALL$Region)       1 145.88
## - as.factor(acousticALL$Echosounder)  1 158.10
## 
## Step:  AIC=143.83
## acousticALL$nTotal ~ as.factor(acousticALL$Echosounder) + as.factor(acousticALL$Region) + 
##     as.factor(acousticALL$HabitatType) + offset(log(acousticALL$TracklineDist.nmi))
## 
##                                      Df    AIC
## - as.factor(acousticALL$HabitatType)  1 141.84
## <none>                                  143.83
## - as.factor(acousticALL$Region)       1 144.05
## - as.factor(acousticALL$Echosounder)  1 158.53
## 
## Step:  AIC=141.85
## acousticALL$nTotal ~ as.factor(acousticALL$Echosounder) + as.factor(acousticALL$Region) + 
##     offset(log(acousticALL$TracklineDist.nmi))
## 
##                                      Df    AIC
## <none>                                  141.84
## - as.factor(acousticALL$Region)       1 142.05
## - as.factor(acousticALL$Echosounder)  1 156.91
```

```
ALLnegBStep$anova #step-wise model selection
```

```
## Stepwise Model Path 
## Analysis of Deviance Table
## 
## Initial Model:
## acousticALL$nTotal ~ as.factor(acousticALL$Echosounder) + as.factor(acousticALL$Region) + 
##     as.factor(acousticALL$HabitatType) + as.factor(acousticALL$SeaState) + 
##     offset(log(acousticALL$TracklineDist.nmi))
## 
## Final Model:
## acousticALL$nTotal ~ as.factor(acousticALL$Echosounder) + as.factor(acousticALL$Region) + 
##     offset(log(acousticALL$TracklineDist.nmi))
## 
## 
##                                   Step Df   Deviance Resid. Df Resid. Dev
## 1                                                           28   33.54573
## 2    - as.factor(acousticALL$SeaState)  1 0.05772384        29   33.60345
## 3 - as.factor(acousticALL$HabitatType)  1 0.05296870        30   33.55048
##        AIC
## 1 145.8289
## 2 143.8335
## 3 141.8455
```

```
summary(ALLnegBStep)
```

```
## 
## Call:
## glm.nb(formula = acousticALL$nTotal ~ as.factor(acousticALL$Echosounder) + 
##     as.factor(acousticALL$Region) + offset(log(acousticALL$TracklineDist.nmi)), 
##     init.theta = 0.7794057841, link = "log")
## 
## Deviance Residuals: 
##     Min       1Q   Median       3Q      Max  
## -1.9468  -1.0089  -0.6527   0.3866   1.8575  
## 
## Coefficients:
##                                     Estimate Std. Error z value Pr(>|z|)
## (Intercept)                          -1.7830     0.3828  -4.658 3.19e-06
## as.factor(acousticALL$Echosounder)1  -2.9338     0.5500  -5.334 9.60e-08
## as.factor(acousticALL$Region)SNE     -0.8092     0.5008  -1.616    0.106
##                                        
## (Intercept)                         ***
## as.factor(acousticALL$Echosounder)1 ***
## as.factor(acousticALL$Region)SNE       
## ---
## Signif. codes:  0 '***' 0.001 '**' 0.01 '*' 0.05 '.' 0.1 ' ' 1
## 
## (Dispersion parameter for Negative Binomial(0.7794) family taken to be 1)
## 
##     Null deviance: 68.484  on 32  degrees of freedom
## Residual deviance: 33.550  on 30  degrees of freedom
## AIC: 143.85
## 
## Number of Fisher Scoring iterations: 1
## 
## 
##               Theta:  0.779 
##           Std. Err.:  0.321 
## 
##  2 x log-likelihood:  -135.845
```

```
#dropterm
ALLdrop<- dropterm(ALLnegB, test= "Chisq") 
ALLdrop
```

```
## Single term deletions
## 
## Model:
## acousticALL$nTotal ~ as.factor(acousticALL$Echosounder) + as.factor(acousticALL$Region) + 
##     as.factor(acousticALL$HabitatType) + as.factor(acousticALL$SeaState) + 
##     offset(log(acousticALL$TracklineDist.nmi))
##                                    Df    AIC     LRT   Pr(Chi)    
## <none>                                145.83                      
## as.factor(acousticALL$Echosounder)  1 158.10 14.2756 0.0001579 ***
## as.factor(acousticALL$Region)       1 145.88  2.0489 0.1523198    
## as.factor(acousticALL$HabitatType)  1 143.84  0.0070 0.9334953    
## as.factor(acousticALL$SeaState)     1 143.83  0.0046 0.9460462    
## ---
## Signif. codes:  0 '***' 0.001 '**' 0.01 '*' 0.05 '.' 0.1 ' ' 1
```

```
ALLnegBminusSS<- glm.nb(acousticALL$nTotal~as.factor(acousticALL$Echosounder)+as.factor(acousticALL$Region)+as.factor(acousticALL$HabitatType)+offset(log(acousticALL$TracklineDist.nmi)), link= "log")
ALLnegBminusSSHab<- glm.nb(acousticALL$nTotal~as.factor(acousticALL$Echosounder)+as.factor(acousticALL$Region)+offset(log(acousticALL$TracklineDist.nmi)), link= "log")
ALLnegBminusSSHabReg<- glm.nb(acousticALL$nTotal~as.factor(acousticALL$Echosounder)+offset(log(acousticALL$TracklineDist.nmi)), link= "log")
```

From a backwards step-wise selection process, Month and Echosounder remain in the model. When using a single term deletion method, only Echosounder remains significant at the 0.05 level (pEcho= 1.579009810^{-4})

Now to test the other model assumptions for the reduced model.

### VIFs

```
vif(ALLnegBStep)
```

```
## as.factor(acousticALL$Echosounder)      as.factor(acousticALL$Region) 
##                           1.001216                           1.001216
```

Once again, all VIFs in the reduced Echosounder + Region model ~1 which indicates no collinearity.

### Linearity on the function’s link scale

```
par(mfrow=c(1,2))
plot(acousticALL$nTotal,fitted(ALLnegBStep), xlab= "Observed values", ylab= "Fitted values", main= "Linearity on the log link scale")
abline(0,1)
#scaling Pearson residuals to take into account overdispersion
phi<- summary(ALLnegBStep)$theta
fits<- fitted(ALLnegBStep, type= "response")
scaledresid<- (acousticALL$nTotal-fits)/sqrt(phi*fits)
plot(fits,scaledresid, xlab= "Fitted values", ylab= "Scaled residuals", main= "Model fit-residuals")
abline(h=0)
```

The model residuals show a slight pattern, checking other model assumptions to see why that is the case.

### Influence

```
par(mfrow=c(1,1))
plot(cooks.distance(ALLnegBStep),main= "Influence of observations") #Cook's distance < 1, pass
```

There does not appear to be any gross influence by any of the observations.

### Testing for independence

```
acf(ALLnegBStep$residuals, main= "Independence of model residuals") #within CI, pass
```

There is no violation of the independence assumption.

### Confidence intervals for covariates

```
confint(ALLnegBStep)
```

```
## Waiting for profiling to be done...
```

```
##                                         2.5 %     97.5 %
## (Intercept)                         -2.435982 -0.9919312
## as.factor(acousticALL$Echosounder)1 -4.117819 -1.8226390
## as.factor(acousticALL$Region)SNE    -1.821709  0.2208893
```

### Test of contrasts: Understanding the relationship between echosounder and region covariates

```
ALLnegBMeans<-interactionMeans(ALLnegBStep)
ALLnegBMeans
```

```
##   as.factor(acousticALL$Echosounder) as.factor(acousticALL$Region)
## 1                                  0                       Georges
## 2                                  1                       Georges
## 3                                  0                           SNE
## 4                                  1                           SNE
##   adjusted mean SE of link
## 1    12.5128162  0.3827816
## 2     0.6656070  0.5133508
## 3     5.5706498  0.3940133
## 4     0.2963252  0.5398626
```

```
plot(ALLnegBMeans)
```
